# Ultrasound radiomics predicts preoperative axillary lymph node metastasis status in early-stage breast cancer to support surgical decisions: a machine learning, monocenter study

**DOI:** 10.3389/fonc.2025.1680160

**Published:** 2026-01-16

**Authors:** Zhi-Liang Hong, Xiao-Rui Peng, Xia Liang, Xian-Tao Zeng, Jian-Chuan Yang, Song-Song Wu

**Affiliations:** 1 Shengli Clinical Medical College of Fujian Medical University, Fuzhou, China; 2 Department of Ultrasound, Fujian Provincial Hospital, Fuzhou, China; 3 Fuzhou University Affiliated Provincial Hospital, Fuzhou, China; 4 Fujian Medical University, Fuzhou, China

**Keywords:** axillary lymphnode metastasis (ALNM), breast cancer (BC), clinical parameters, clinical stages T1–2, machine learning(ML), radiomics

## Abstract

**Background:**

The usual assessment for axillary lymph node (ALN) status in breast cancer (BC) in current clinical practice is based on an invasive procedure that has a low efficiency rate and frequently results in operative-associated problems for patients. Therefore, our goal was to create an effective preoperative ultrasound (US) radiomics evaluation method for ALN status in patients with clinical stages T1–2 invasive BC using machine learning (ML) approaches.

**Methods:**

Between January 2020 and January 2024, we retrospectively analyzed the medical records of 671 patients with histologically proven malignant breast tumors in our hospital.The data set was divided into model training group and validation testing group with a 75/25 split.There were two categories for ALN tumor burden: low (1–2 metastatic ALNs) and high (≥ 3 metastatic ALNs). The PyRadiomics package was used to obtain radiomic features (RF), and a support vector machine (SVM) with the LASSO approach was used to create a radiomic signature (RS).The training group’s multivariate logistic regression results were used to create a nomogram that combined the BC US radiomics score with a clinical parameter.Additionally, the area under the operating characteristic curve (AUC) was used to evaluate their prediction performance.

**Results:**

With an AUC of 0.920 (95% CI: 0.901, 0.943) in the test cohort, clinical parameter coupled RS provides the greatest diagnostic performance in predicting ALN status between disease-free axilla and any axillary metastases.In the testing cohort, the sensitivity, specificity, accuracy, positive predictive value (PPV), and negative predictive value (NPV) were 90%, 82%, 83%, 89%, and 86%, respectively. With an AUC of 0.939 (95% CI: 0.892, 0.970) in the test cohort, this clinical measure paired with RS can also distinguish between a low and a substantial metastatic burden of axillary illness.

**Conclusions:**

For patients with early-stage BC, our work provides a noninvasive imaging biomarker to forecast the extent of ALN metastases.The imaging biomarker demonstrated strong predictive value and the potential for extended application to customize surgical care.

## Introduction

Globally, female breast cancer (BC) is the main cause of cancer-related mortality for women and has overtaken lung cancer as the most often diagnosed malignancy (1). One of the key prognostic variables for BC that influences treatment choices is axillary lymph node metastasis (ALNM) (2). The first node to drain the main malignancy is the sentinel lymph node (SLN).To anticipate ALN status, SLN dissection (SLND) is advised, particularly for patients with clinically negative nodes (3). According to the American College of Surgeons Oncology Group Z0011 (ACOSOG Z0011) trial, SLND by itself would not result in a lower survival rate than ALND in patients with clinical T1/T2 BC if there were two or less SLN metastases (4, 5). Although SLND has fewer difficulties than ALND, it is still a risky procedure with several serious drawbacks, such as a significant increase in anesthetic time and cost and the possibility of complications such upper limb edema or arm numbness in 3.5% to 10.9% of patients (6, 7).Additionally, radiopharmaceuticals and the methods of injecting radiocolloid limit its accuracy (8, 9). Additionally, the frozen segment of SLNB takes a long time, which prolongs the duration of the procedure.According to some research, 43–65% of patients with positive SLNs had needless axillary surgery because there was no nonSLN metastases, which led to a high rate of morbidity (10, 11). Because the majority of patients with early-stage BC (cT1-2N0M0) have disease-free axilla, SLN biopsy may be avoided if there was a trustworthy and effective preoperative assessment of ALN status (12).

To assess ALN status and describe breast lesions preoperatively, ultrasound (US) has been employed extensively (13). In patients with early-stage BC, a study found that preoperative axillary US results and clinical T stage were related to the ALN status (12). However, axillary US had poor diagnostic performance in determining the ALN status, with an area under the receiver operating characteristic curve (AUC) of 0.585–0.719 (14). Numerous investigations aimed to predict the ALN status using clinicopathological information, including hormone receptor status, tumor grade, histological tumor size, lymphovascular invasion, and Ki-67 proliferation index (15, 16). However, with an AUC of 0.66–0.74 in earlier research, relying solely on clinicopathological data is insufficiently accurate (17). Furthermore, preoperative information of ALN status is crucial for choosing the best axillary treatment options, even though some data, such as lymphovascular invasion and histological tumor size, may not be available (6).

Many quantitative picture elements that are typically difficult for the human eye to identify can be automatically extracted from medical images using radiomics (18, 19). Subsequent examination of these characteristics may reveal intratumor heterogeneity and offer prospective noninvasive biomarkers to aid in clinical decision-making (18, 20).Clinical oncology CT or MRI image analysis was the first application of this technique that showed promise (21, 22). Radiomics based on US image analysis has recently outperformed other standard techniques (23). Through supervised learning, a recently developed technique called deep learning radiomics (DLR) can extract quantitative and high-throughput features from medical images (18, 19). When DLR is used to evaluate medical images, it usually has small-sample learning problems.In order to improve model performance, clinical parameter combined DLR, which combines clinical information with network characteristics, can assist in providing complementary information for image features and working together to create the model using clinical information and US image features (24).To date, radiomics and deep learning have been used less frequently to forecast ALNM in BC.

The objective of this study was to develop a novel preoperative strategy based on US radiomics that focuses on tumor regions for ALNM prediction in patients with early-stage invasive BC. The ultimate goal is to assist in customizing surgical management and minimizing needless SLNB or ALND, which will lower the incidence of related postoperative complications and enhance patients’ quality of life.

## Methods

### Patients

The Fujian Provincial Hospital review committee gave their approval to this study. All participants provided informed consent to participate in the study.The study adhered to the Declaration of Helsinki and relevant national guidelines.All experiments were performed in accordance with relevant guidelines and regulations.We constantly gathered female patients having US findings of breast lesions from January 2020 to January 2024.The following were among the requirements for inclusion: (a) women with US-suspected breast masses; (b) clinical data availability; (c) patients who had breast surgery and SLNB or ALND with the goal of curing their condition; and (d) each patient only had a lesion with a diameter of less than or equal to 5 cm (stages T1 and T2), as determined by pathological results and US imaging. The following conditions were excluded: (a) preoperative treatment (chemotherapy, neoadjuvant radiation, or resection biopsy); (b) individuals with bilateral disease or multifocal lesions; (c) the region of interest (ROI) was not fully apparent on the US images;(d) benign breast lesions or cancer *in situ*; (e) missing significant histopathological results (immunohistochemical or LN results); (f) insufficient information or images. In accordance with the NCCN guideline, imaging (MRI, ultrasound, or positron emission tomography) or clinical examination were used to assess the clinical T and clinical N stages (25). SLNB or ALND was used to pathologically determine the ALN state, which was the main conclusion.Lastly, 671 lesions from 671 patients with invasive BC in its early stages were included.The data were divided 75/25 using SPSS version 20.0’s random sample techniques (SPSS, Chicago, IL, USA); 75% of the cases were allocated to the training group. which was used to establish the evaluation system, and 25% of the cases formed the validation group, which was used to test the accuracy of the model prediction.

### Conventional US examinations

All of the preoperative breast and axillary US were examined and assessed by the same radiologist (W.SS) with over 20 years of experience of breast US examination. The radiologist performed with Philips EPIQ7 equipped with a L12–5 linear array probe. The target breast mass was measured at maximal-diameter plane to determine US size and classified by using US BI-RADS (26).The radiologist recorded worrisome US findings of ALN and performed routine axillary US after doing whole-breast US. The following are suspicious US features of ALN: nonhilar cortical blood flow on color Doppler images; rounded hypoechoic node; complete or partial effacement of the fatty hilum; diffuse cortical thickening>3 mm; focal cortical bulge>3 mm; eccentric cortical thickening>3 mm; complete or partial replacement of the node with an ill-defined or irregular mass; and microcalcifications in the node (27). As long as at least one questionable US finding was discovered, the skilled radiologist’s evaluation of the axillary US was considered positive. When there were no worrisome ALN results, the axillary US test was considered negative (12).

### Clinical characteristics

After SLN biopsy and initial tumor resection, all patients had ALND on anatomic levels I and II with at least 10 nodes removed (4, 28). After undergoing histological analysis, all of the removed ALNs were categorized as either metastatic or nonmetastatic nodes (29). The pathology reports of the patients were reviewed in order to obtain clinical characteristics such as the number of metastatic LNs, the status of human epidermal growth factor receptor 2 (HER2), the estrogen receptor (ER), the progesterone receptor (PR), the KI-67 levels, the histological tumor type, and the LN status (LN with macrometastasis or micrometastasis was considered positive).If 10% or more of the tumor’s cells were immunostained, the tumor was deemed ER or PR positive. At least 3+ hematoxylin-eosin (HE) staining indicates HER2 positivity, while at least 14% of immunostained cells indicate KI-67 positivity. The expression of ER, PR, and HER-2 status were used to categorize the molecular subtype of BC (30). Each patient’s pathologic SLN (pSLN) load was divided into two groups: those with ≥3 metastatic SLNs and those with 1–2 metastatic SLNs.Clinical information from patient medical records, including age and physical examination results, was gathered.

### US data processing and analysis

**Figure 1:**
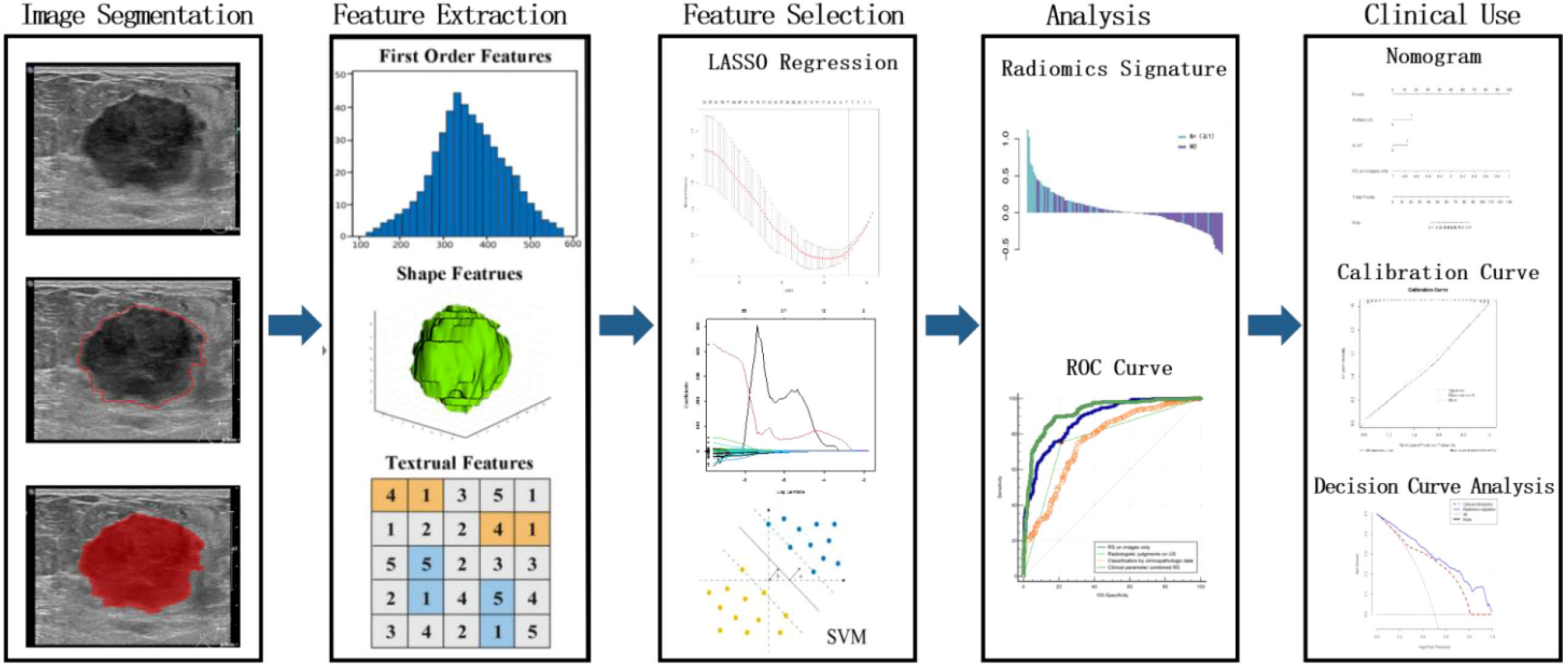
The workflow of the critical steps. Tumors are segmented manually on Two-dimensional ultrasound image. Radiomic features are extracted to quantify tumor intensity, shape, and texture within the defined tumor contours. The least absolute shrinkage and selection operator (LASSO) method and Support vector machine are jointly applied to feature selection. The radiomics signature is constructed by a linear combination of selected features, and the performance of related radiomics nomogram is assessed by the area under the receiver operating characteristic (ROC) and calibration curves, followed by a decision curve analysis.

Image segmentation, high-throughput feature extraction, feature selection, predictive model building, model evaluation, validation, and clinical application comprised the flowchart (**Figure 1**) that depicted the radiomic approach for predicting LN metastasis.

### Definition of the ROI

Two radiologists, H.ZL, who had over 7 years of experience with breast US examinations, and W.SS, who had 25 years of expertise with breast US, chose the US images and were in charge of defining the ROI. The clinical and pathological details of the patients were concealed from the two radiologists. The ROI should, to the greatest extent feasible, include the whole lesion and was drawn along the BC’s edge on a few representative pictures of each patient. At last, a picture of the entire tumor was acquired.The radiologist (H.ZL) drew the ROIs using ITK-SNAP software (http://www.itksnap.Org/pmwiki//pmwiki.php). Another radiologist (W.SS) drew the ROIs in 100 randomly chosen lesions in order to assess inter-observer variability. A week later, H.ZL. performed the second delineation of ROIs from 100 randomly selected photos using the same process in order to evaluate intraobserver reliability. The intraclass correlation coefficient (ICC) was used to quantify the intraobserver agreement and interobserver consistency of ROI as depicted by two radiologists. ICC > 0.75 is seen as a sign of strong consistency.

### Radiomics feature extraction and selection

To extract RF from the US photos, we used an open-source Python tool named “PyRadiomics” (31), and all of the outcomes were compiled into a form. The training cohort underwent a two-stage feature selection process to eliminate redundant and unnecessary information. Features with p < 0.1 were chosen as potentially informative features after Mann-Whitney U tests were conducted.The best features were then chosen using two selection models. The first model, a least absolute shrinkage and selection operator (LASSO) regression model, uses the regulation parameter λ in the training cohort to reduce the coefficients of irrelevant features to zero. To determine the ideal regulation parameter for λ using the 1-SE rule, a second model (the leave-one-out cross-validation (LOOCV) approach) was employed. Therefore, the most representative features were those that had a non-zero coefficient in the model with an ideal regulation parameter for λ. A minimum of 15 observations were needed for each predictor variable in order to construct a suitably stable model. Furthermore, the most valuable predictive features were chosen using a recursive feature elimination (RFE) estimator that gives features weights. Sequential forward selection (SFS) was used when models chose too many features in order to prevent over-fitting. Python software called “scklearn” (Python Foundation for Statistical Computing), Version 2.7, was used to create selection models.

### Prediction nomogram construction

#### Radiomic signature

The support vector machine (SVM) algorithm was used to create the signature using radiomics features chosen using the LASSO approach. The ideal regulation parameter “C,” which was intended to strike a balance between the requirement for diagnostic accuracy and avoiding an unduly complex model in the training cohort, was found using sixfold validation. The RS based on the chosen features was fitted to the training cohort and then further validated in the validation cohort after the ideal value for C was determined.

#### Clinical model

Univariate analysis were used to evaluate the relationship between LN metastases and traditional clinical risk variables. Multivariate analysis employed forward selection logistic regression (LR) to construct the clinical model in the training cohort based on the clinical characteristics with p < 0.05 in the univariate analyses. The validation set was then used to confirm the clinical model’s performance.

#### Combined model

The LR approach was used to build the combined model, which included the clinical components in the clinical model and the RF in the best RS. The validation cohort was then used to confirm the combined model’s performance. The performance of the aforementioned models was assessed using the receiver operating characteristic (ROC) curve and AUC. The combined model and the clinical model were compared using the Delong test based on AUC values.

#### Nomogram establishment

A nomogram for the clinical and combination models was generated in order to give patients and physicians a personalized and user-friendly tool for predicting LN metastases. A calibration curve was used to evaluate how well the LN metastasis projections and the actual results agreed. Additionally, the clinical and combined nomogram’s performance was evaluated using the Hosmer–Lemeshow test.

By measuring the net benefits at various threshold probabilities, decision curve analysis (DCA) was used to assess the RF’s clinical utility.

### Statistical analysis

The statistical analysis was conducted using SPSS 22.0 (IBM Corp., Armonk, NY) and R software (version 4.2.1; https://www.r-project.org/). Radiomics analysis was performed using Python 2.7.Continuous variables having a normal distribution were compared using the Student’s t-test. When comparing continuous variables with an uncertain or aberrant distribution, the Mann-Whitney U test was employed. Categorical variables were compared using the χ² test. Statistical significance was defined as P values below 5%.According to the ICC, the two radiologists’ intra-observer agreement and inter-observer consistency in ROI delineation were rated as very good (0.80 to 1.00), good (0.60 to 0.80), fair (0.40 to 0.60), moderate (0.20 to 0.40), or poor (< 0.20).ROC analysis was used to evaluate the signatures’ prediction accuracy. The sensitivity and specificity of each signature were assessed using the area under the ROC curve (AUC). Furthermore, DCA was used to quantify the net benefits when various threshold probabilities were taken into account in order to evaluate the prediction model’s clinical utility.

## Results

### Baseline characters

**Figure 2:**
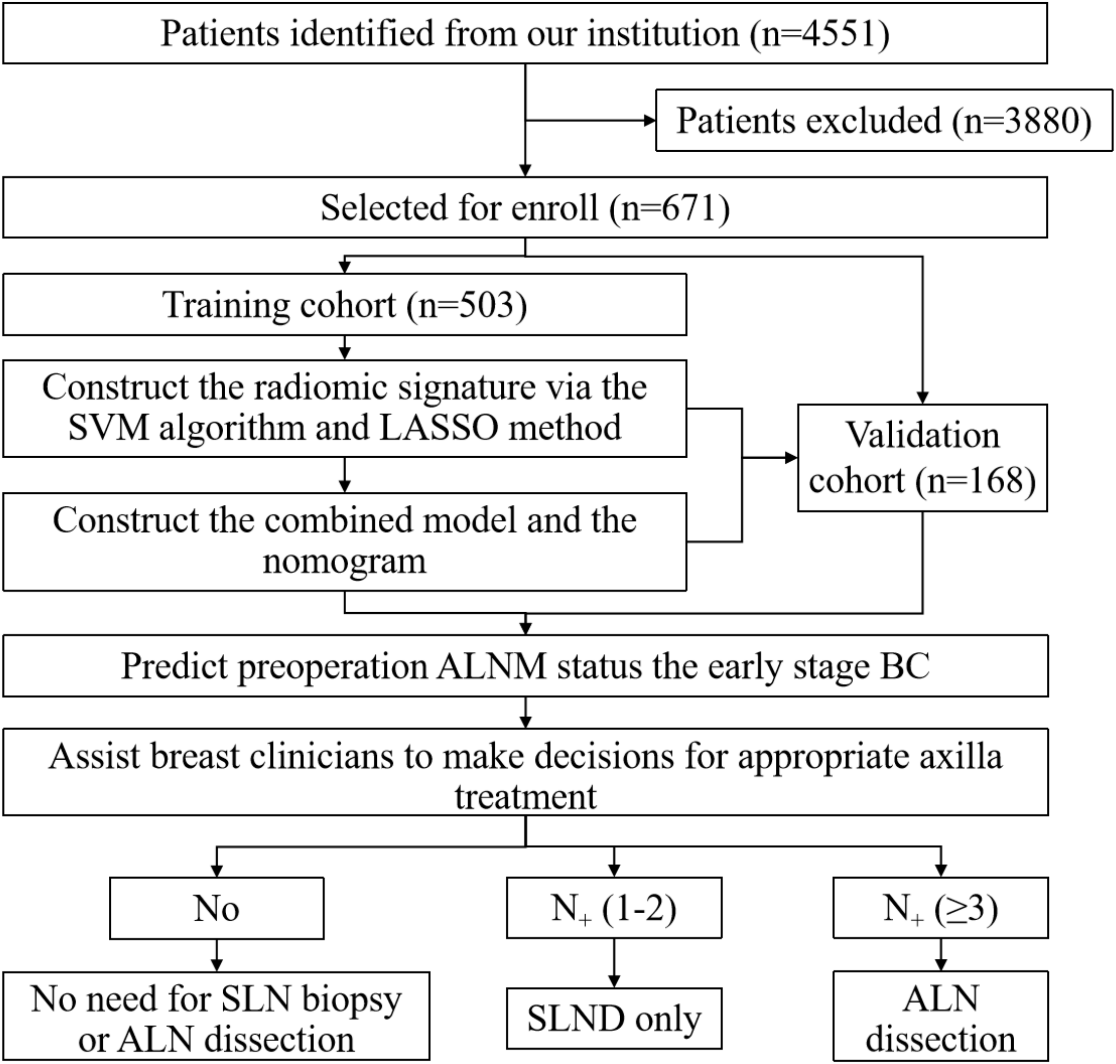
Patient recruitment workflow. In total, 671 out of 4551 patients were included according to the selection criteria. The included patients were examined by conventional US, and had complete clinical information needed for the study.

938 of the 4,551 women who were recruited in the research received a BC diagnosis following surgery. 671 women were ultimately included following a thorough screening process based on our inclusion and exclusion criteria. 503 people were chosen at random to be in the training group. The patient recruitment process is depicted in **Figure 2**. 256 had disease-free axilla (N0), 75 had mild metastatic burden of axillary disease (N+(1–2)), and 172 had heavy metastatic burden of axillary disease (N+(≥3)), based on the results of SLND or ALN dissection. **Table 1** provides a summary of each patient’s clinicopathologic features. There is no statistically significant difference in the clinical characteristics of the patients in the training and validation sets.It was shown that the radiologists’ judgments of US, US size, and Ki-67 status were independent predictors of BC (P<0.001).An ICC of 0.799 ± 0.022 indicated significant inter-observer consistency in the RF extraction between the two radiologists, whereas an ICC of 0.839 ± 0.019 indicated strong intra-observer reliability.

**Table 1:** Clinicopathologic characteristics between axillary lymph node positive and negative groups in the training and validation cohorts.

Characteristic	Training cohort			Validation cohort			p
	(No. of patients[n] = 503)			(No. of patients[n] = 168)			
	Axillary lymph node status			Axillary lymph node status			
	Negative (n = 256)	Positive (n = 247)	p	Negative (n = 87)	Positive (n = 81)	p	
Age, mean ± SD, years	52.54 ± 11.31	51.70 ± 11.21	0.406	51.99 ± 12.31	52.15 ± 12.46	0.934	0.961
US size,mean ± SD, mm	22.89 ± 11.82	33.35 ± 12.26	<0.001	24.00 ± 12.56	34.34 ± 12.86	<0.001	0.419
Menopausal status(%)			0.826			0.414	0.207
Yes	58 (22.7)	58(23.5)		14(16.1)	17(21.0)		
no	198(77.3)	189(76.5)		73(83.9)	64(79.0)		
BC laterality(%)			0.364			0.929	0.531
Left	143(55.9)	128(51.8)		50(57.5)	46(56.8)		
Right	113(44.1)	119(48.2)		37(42.5)	35(43.2)		
Tumor location(%)			0.08			0.632	0.453
UOQ	145(56.6)	150(60.7)		54(62.1)	53(65.4)		
LOQ	26(10.2)	35(14.2)		6(6.9)	8(9.9)		
UIQ	52(20.3)	43(17.4)		14(16.1)	12(14.8)		
LIQ	31(12.1)	15(6.1)		12(13.8)	6(7.41)		
Central	2(0.78)	4(1.62)		1(1.15)	2(2.47)		
BI-RADS category(%)			<0.001			<0.001	0.862
4A category	26(10.2)	5(2.02)		11(12.6)	2(2.47)		
4B category	56(21.9)	12(4.86)		17(19.5)	5(6.17)		
4C category	87(33.9)	65(26.3)		31(35.6)	16(19.8)		
5 category	87(33.9)	165(66.8)		28(32.2)	58(71.6)		
Radiologists’ judgments on US(%)			<0.001			<0.001	0.999
Positive	45(17.6)	204(82.6)		12(13.8)	71(87.7)		
Negative	211(82.4)	43(17.4)		75(86.2)	10(12.3)		
Tumor type(%)			0.086			0.023	0.816
Invasive ductal carcinoma	178(69.5)	193(78.1)		59(67.8)	69(85.2)		
Invasive lobular carcinoma	10(3.91)	6(2.43)		4(4.60)	3(3.70)		
Other tumor types	68(26.6)	48(19.4)		24(27.6)	9(11.1)		
ER(%)			0.345			0.961	0.899
Positive	186(72.7)	170(68.8)		62(71.3)	58(71.6)		
Negative	70(27.3)	77(31.2)		25(28.7)	23(28.4)		
PR(%)			0.334			0.502	0.316
Positive	158(61.7)	142(57.5)		46(52.9)	47(58.0)		
Negative	98(38.3)	105(42.5)		41(47.1)	34(42.0)		
HER2(%)			0.037			0.422	0.857
Positive	54(21.1)	72(29.1)		20(23.0)	23(28.4)		
Negative	202(78.9)	175(70.9)		67(77.0)	58(71.6)		
Ki-67(%)			<0.001			0.003	0.281
Positive	192(75.0)	225(91.1)		61(70.1)	72(88.9)		
Negative	64(25.0)	22(8.90)		26(29.9)	9(11.1)		
Molecular subtypes (%)			0.218			0.869	0.867
Luminal A	163(63.7)	139(56.3)		52(59.8)	46(56.8)		
Luminal B	24(9.36)	32(13.0)		10(11.5)	12(14.8)		
HER2-positive	30(11.7)	40(15.2)		10(11.5)	11(13.6)		
Triple negative	39(15.2)	36(14.6)		15(17.2)	12(14.8)		

Qualitative variables are in n (%) and quantitative variables are in mean ± SD, when appropriate.

US: ultrasound; BC, breast cancer; UIQ, upper inner quadrant; UOQ, upper outer quadrant; LOQ, lower outer quadrant; LIQ, lower inner quadrant.

### Construction of the radiomics signature and radiomic nomogram

**Figure 3:**
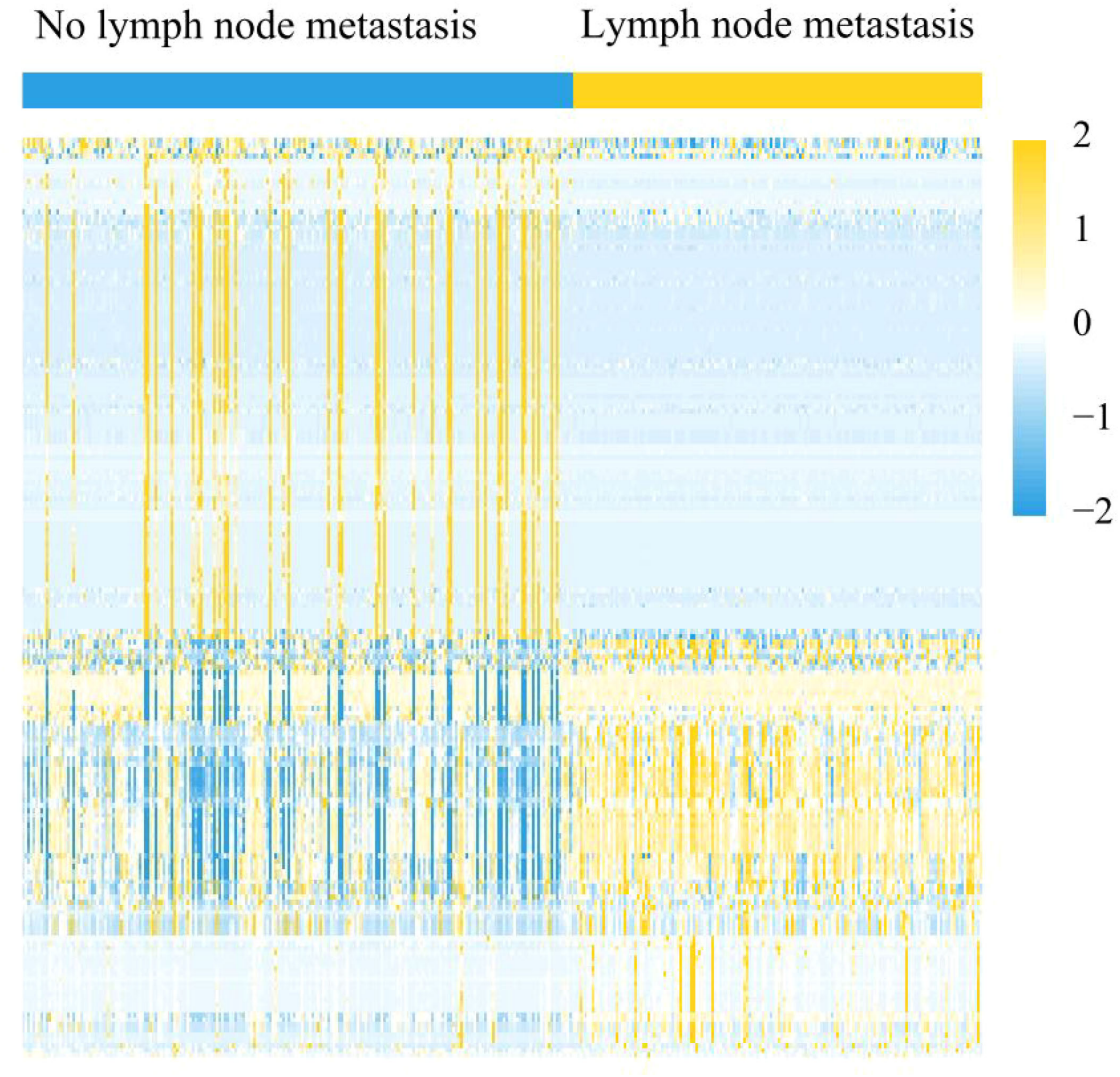
Overall distribution of key radiomic features from US image among patients with and without ALN metastasis in the training cohort.

The training cohort’s overall distribution of important radiomic markers between patients with and without ALNM is shown in **Figure 3**, clearly highlighting the differences between the two groups. The formula for the radiomics score took these characteristics into account.Both the training (P < 0.0001) and testing (P < 0.001) cohorts showed a statistically significant difference in rad score between the LNM group (N+(≥1)) and the non-LNM group (N0). **Figure 1** displays the radiomics signatures of patients with and without ALNM.With an AUC of 0.878 in the training cohort and 0.876 in the testing cohort, the RS demonstrated strong predictive efficacy.

Based on the RS, the radiologists’ assessments of the US, and the Ki-67 status, we created a nomogram (**Figure 1**). The Hosmer-Lemeshow test was used to examine the nomogram’s calibration curve, and the results showed that it was well calibrated (**Figure 1**) with non-significant results (p = 0.21 and 0.35 in training and validation cohorts, respectively).

### Prediction of ALN status between N0 and N_+_(≥1).

Using N0 as the negative reference standard, 168 lesions were designated as the test cohort and 503 lesions as the training cohort at random. **Table 2** provided an example of the specific attributes. An expert radiologist reviewed the axillary US data, which showed an AUC of 0.765, accuracy of 0.756, sensitivity of 0.745, and specificity of 0.776. For axillary US, the intra-observer agreement was 0.977 and the inter-observer agreement was 0.924 (both P < 0.001, Kappa test).We discovered that the RS outperformed radiologists’ diagnoses on the same US in terms of accuracy, AUC, and sensitivity when comparing the performance of the ALN status prediction between N0 and N+(≥1) between the RS and radiologists’ assessments on US in the training and the validation cohorts. (**Table 3**).

**Table 2:** The prediction of ALN status results (N0 v.s. N+(≥1)).

Methods	Group	SENS	SPEC	ACC	PPV	NPV	AUC (95% CI)
Classification by clinicopathologic data	T	0.75	0.70	0.72	0.70	0.74	0.76(0.72-0.80)
	V	0.75	0.63	0.69	0.66	0.73	0.74(0.67-0.81)
ALN RS	T	0.76	0.83	0.80	0.81	0.78	0.88(0.85-0.91)
	V	0.88	0.73	0.80	0.75	0.87	0.88(0.82-0.93)
Clinical parameter combined RS	T	0.90	0.82	0.86	0.83	0.89	0.92(0.90-0.94)
	V	0.94	0.82	0.88	0.82	0.93	0.94(0.89-0.97)

95% confidence intervals are included in brackets. AUC area under the receiver operating characteristic curve; ACC accuracy; SENS sensitivity; SPEC specificity; PPV positive predict value; NPV negative predict value, T training cohort (n = 503); V validation cohort (n = 168). ALN axillary lymph node;RS radiomic signature.

**Table 3:** Comparison of the performance for ALNM prediction between the RS and radiologists’ judgments on US.

Cohort	Signature	Signature performance					
		SENS	SPEC	ACC	PPV	NPV	AUC (95%CI)
Training cohort	ALN RS	0.76	0.83	0.80	0.81	0.78	0.88(0.85-0.91)
	Radiologists’ judgments on US	0.75	0.78	0.76	0.77	0.76	0.77(0.73-0.81)
validation cohort	ALN RS	0.88	0.73	0.84	0.75	0.87	0.88(0.82-0.93)
	Radiologists’ judgments on US	0.67	0.86	0.74	0.82	0.73	0.76(0.69-0.83)

US, ultrasound; ALN, axillary lymph node; PPV, positive predictive values; NPV, negative predictive values; CI, confidence interval; AUC, area under the receiver operating characteristics curve; ACC accuracy; SENS sensitivity; SPEC specificity.

Additionally, we gathered data on radiologists’ assessments of 400 patients’ US and MRI pictures from the Jinshan branch of Fujian Provincial Hospital. We then contrasted the performance of the RS and radiologists’ assessments based on both US and MRI images. The findings showed that the RS had improved accuracy, specificity, and sensitivity. The RS’s clinical value outperformed that of conventional clinical practice. (**Table 4**).

**Table 4:** Comparison of the performance for ALNM prediction between ALN RS and radiologists’ judgments on both MRI and US.

Signature	Signature performance					
	SENS	SPEC	ACC	PPV	NPV	AUC (95% CI)
ALN RS	0.77	0.83	0.81	0.80	0.80	0.87(0.84-0.92)
Radiologists’ judgments on MRI	0.73	0.79	0.75	0.78	0.74	0.76(0.71-0.83)
Radiologists’ judgments on US	0.73	0.77	0.74	0.77	0.74	0.75(0.70-0.82)

MRI, magnetic resonance imaging; ALN, axillary lymph node; PPV, positive predictive values; NPV, negative predictive values; CI, confidence interval; AUC, area under the receiver operating characteristics curve; ACC accuracy; SENS sensitivity; SPEC specificity.

**Figure 4:**
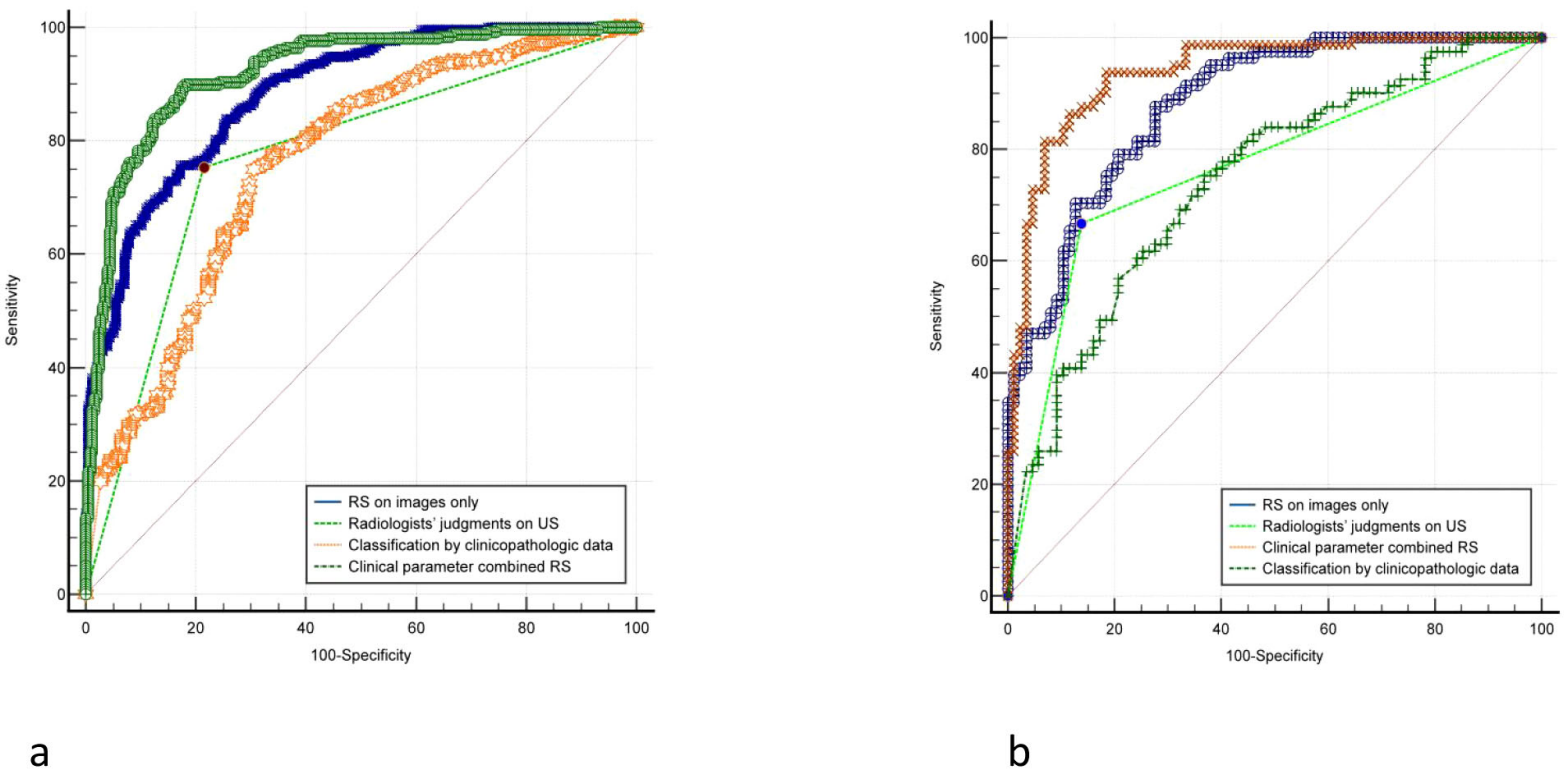
Comparison of receiver operating characteristic (ROC) curves between different models for predicting disease-free axilla (N0) and any axillary metastasis (N+(≥1)). **(A)** The ROC of training group. **(B)** The ROC of validation group.

While RS based solely on pictures and classification by clinicopathologic data only obtained AUCs of 0.878 and 0.759, respectively, clinical parameter combination RS attained the highest AUC of 0.921 in the training cohort. AUCs for predicting ALNM in the independent test cohort decreased little, but they remained consistent with the training cohort’s performance. The maximum AUC of 0.938 was still attained by the clinical parameter combination RS, surpassing the AUC of other approaches such as classification by clinicopathologic data (AUC:0.741) and RS (AUC:0.875). Additionally, the clinical parameter combined RS’s accuracy, sensitivity, specificity, PPV, and NPV were consistently superior to those of other techniques. **Table 2** provided a summary of the comprehensive statistical findings, and **Figure 4** displayed the associated ROCs.

### Prediction of ALN status between N_+_(1–2) and N_+_(≥3)

**Figure 5:**
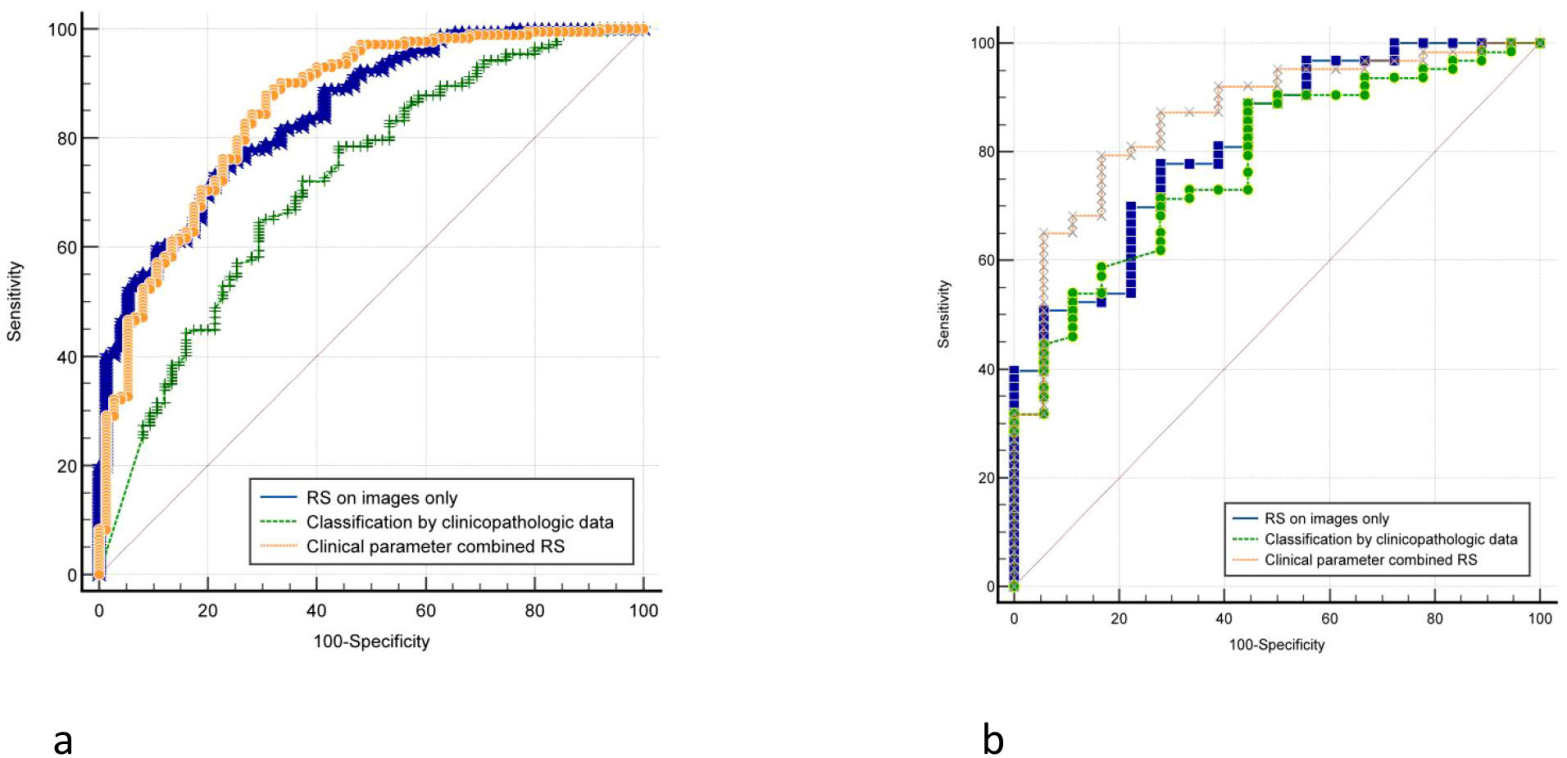
Comparison of receiver operating characteristic (ROC) curves between different models for predicting low ALN burden (N+(1**–**2))and heavy ALN burden (N+(≥3)). **(A)** The ROC of training group. **(B)** The ROC of validation group.

In this experiment, 247 lesions were assigned as the training cohort and 81 as the test cohort, using N+(1–2) as the negative reference standard. In the training cohort, the AUCs for RS based solely on pictures and classification using clinicopathologic data were 0.848 and 0.722, respectively, but the AUC for clinical parameter combination RS was 0.854. The AUC of clinical parameter combination RS increased marginally to 0.873 in the independent test cohort, surpassing both the AUC of categorization by clinicopathologic data (AUC: 0.779) and the AUC of RS (AUC: 0.822). **Table 5** provided a summary of the comprehensive statistical findings. The comparisons were shown by the matching ROCs (**Figure 5**).

**Table 5:** The prediction of ALN status results (N+(1–2) v.s. N+(≥3)).

Methods	Group	SENS	SPEC	ACC	PPV	NPV	AUC (95% CI)
Classification by clinicopathologic data	T	0.65	0.71	0.67	0.73	0.62	0.72(0.66-0.77)
	V	0.88	0.56	0.79	0.70	0.78	0.78(0.57-0.84)
ALN RS	T	0.74	0.79	0.77	0.81	0.71	0.85(0.80-0.89)
	V	0.78	0.72	0.77	0.77	0.73	0.82(0.72-0.90)
Clinical parameter combined RS	T	0.88	0.70	0.83	0.78	0.82	0.85(0.80-0.90)
	V	0.80	0.83	0.80	0.86	0.77	0.87(0.77-0.93)

95% confidence intervals are included in brackets. AUC area under the receiver operating characteristic curve;ACC accuracy; SENS sensitivity; SPEC specificity; PPV positive predict value; NPV negative predict value; T training cohort (n = 247), V validation cohort (n = 81).

### Prediction of ALN status among N0, N+(1–2) and N+(≥3)

**Figure 6:**
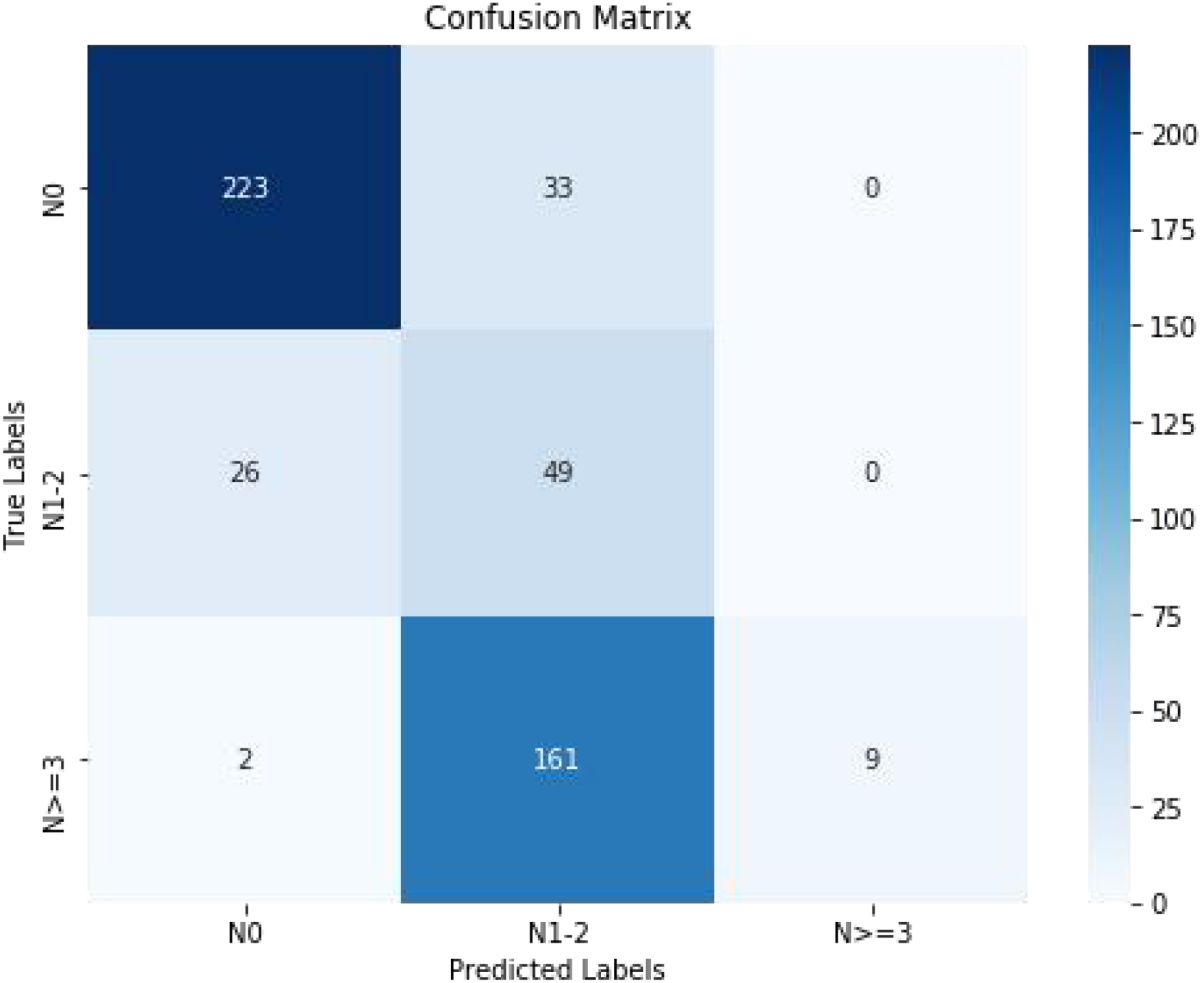
The confusion matrix of predicting ALNM among (N0), [low-load ALNM (N+ (1–2)] and heavy-load ALNM [N+ (≥3)]. ALNM, axillary lymph node metastasis.

In order to forecast ALN status, this model was expanded to work with three different task groups. N0, N+(1–2), and N+(≥3) were the three categories into which the clinical endpoints were divided, as previously mentioned. There are 256 (N0), 75 (N+(1–2)), and 172 (N+(≥3)) lesions in each of the three groups.Breast standard US photos served as the foundation for the RS model. The confusion matrix was displayed in **Figure 6**, and the overall accuracy of distinguishing the three groups was 0.807. While the model did a good job of differentiating the N0 group, it did a worse job in the other two groups.

### Subgroup analysis of the RS for ALNM prediction

Additionally, we performed a subgroup study among patients with varying BI-RADS categories in order to evaluate the RS’s added value to the ALN status. (**Table 6**). The RS was able to detect ALNM patients in the training and validation cohorts’ subgroups of the BI-RADS 4A category (AUC, 0.87, 0.81, respectively), 4B category (AUC, 0.88, 0.87, respectively), 4C category (AUC, 0.85, 0.91, respectively), and 5 category (AUC, 0.92, 0.92, respectively).Additionally, the AUC value of the RS stayed at 0.86-0.92 in these subgroups when stratified by additional variables such US size, Ki67 expression level, and radiologists’ assessments of US.

**Table 6:** Subgroup analysis of the RS for ALNM prediction.

Characteristic	Training cohort	Validation cohort
	(AUC)	(AUC)
US size		
<30	0.89	0.86
≧30	0.87	0.91
Ki67		
Positive	0.87	0.86
Negative	0.88	0.91
Radiologists’ judgments on US(%)		
Positive	0.86	0.87
Negative	0.92	0.91
BI-RADS category		
4A category	0.87	0.81
4B category	0.88	0.87
4C category	0.85	0.91
5 category	0.92	0.92

ALN, axillary lymph node; AUC, area under the receiver operating characteristics curve; HER2, human epidermal growth factor receptor 2; Ki67, proliferation marker protein Ki-67.

### RS for clinical decision-making

Decision curve analysis, which measured the net benefits at different threshold probabilities, was used to evaluate the therapeutic value of the RS. The decision curve showed that the use of the RS to predict ALNM offered more benefits than the clinical risk variables at any threshold probability (**Figure 1**). Because the pathologic ALN state and the clinical ALN status as established by the preoperative US are often different, even senior radiologists can make mistakes in clinical practice. The RS successfully recognized ALNM in patients in both the training and validation groups.

## Discussion

When BC patients are first diagnosed, their ALN state is varied, and their treatment options also vary based on their ALN condition. Patients with positive ALN status have been shown to have worse outcomes than those with negative ALN status (32). According to NCCN guidelines, patients with a positive ALN status are deemed high-risk and require adjuvant chemotherapy, which is crucial for clinicians’ decision-making (25). Neoadjuvant treatment is also being evaluated for certain triple-negative BC patients who have a higher tumor burden and a clinically positive ALN status. It should be mentioned that patients are overtreated and medical resources are wasted when the ALN status is miscalculated. Endocrine therapy alone can be used to treat patients with negative ALN status and no additional risk factors, which results in less expensive and more comfortable treatment.For patients with early-stage BC, it is crucial to correctly and unambiguously distinguish the ALN status.

In order to predict the ALN status prior to surgery in patients with clinical T1 or T2 BC, we created and validated an RS mixed clinical parameter technique based on breast conventional US.Compared to any single method, our approach demonstrated noticeably improved diagnostic results in differentiating between patients with a negative axilla (N0) and those with any axillary metastases (N+(≥1)). Motivatingly, our model demonstrated a good ability to distinguish between individuals with a low axillary disease metastatic load (N+(1–2)) and those with a high burden (N+(≥3)). For patients with early-stage BC, this clinical parameter paired with RS may be able to replace SLND as a noninvasive imaging biomarker with a false-negative rate comparable to SLND. With no requirement for SLN biopsy or ALN dissection in patients with N0, SLND only for patients with N+(1-2), and ALN dissection for patients with N+(≥3), our model demonstrated the potential to help breast doctors make decisions regarding the best axillary treatment (**Figure 1**).Furthermore, in patients with inconsistent clinical and pathologic ALN status, the RS increased the accuracy of ALN status evaluation. Additionally, it correctly identified ALNM in patients with varying BI-RADS categories, US sizes, Ki67 expression levels, and radiologists’ assessments of US. The results showed that the RS is a good tool for ALNM prediction, offering helpful preoperative messages for diagnosis and treatment choices.In individuals with early-stage invasive BC, it can also avoid needless ALND and even SLNB.

Currently, the overall diagnostic effect of preoperative axillary US is low. The axillary US AUC in this study was 0.77, which is in line with findings from earlier research (12, 33, 34).Even senior radiologists can make mistakes in clinical practice, as the pathologic ALN status and the clinical ALN status determined by the preoperative US are frequently not the same. Tumor size, grade, and other tumor-related characteristics have been used to predict LNM in order to minimize the error. The RF obtained from tumor regions for ALNM prediction was investigated in this study because other researchers have demonstrated that the RF derived from tumors with a wealth of biological information may also predict LNM. The “seed and soil” idea states that the synergy between the tumor cells (seed) and the ALN milieu (soil) is what initiates ALNM, and that the LN diagnostic model’s efficacy can be enhanced by the US profiles of the tumor or ALN regions.

Certain lesions’ US features, including tumor size, internal blood supply, lesion boundary, infiltration of subcutaneous adipose tissues, and infiltration of the interstitial adipose tissue breach, have been shown to be independent risk factors for ALNM (35, 36). Furthermore, histological information such as lymphatic vascular invasion, ER, and PR has been found in several research to be a possible risk factor for ALNM (36, 37). Nevertheless, certain histopathological information, such the size of the histological tumor and lymphovascular penetration, could only be assessed following surgical resection and could not inform preoperative choices for axillary surgery. Prior studies have revealed poor concordant rates between core needle biopsy and surgical excision, ranging from 67% to 75%, despite the fact that tumor grade may be determined from core biopsy samples prior to surgery (38, 39).In contrast to the majority of earlier research, all histopathology data included in this study were acquired prior to surgery. Prior to surgery, comprehensive US characteristics of the lesions were gathered. In order to build prediction models that could be fully utilized as non-invasive prediction techniques for determining the ALN status, some preoperative clinical data were kept as candidate factors.

Clinical parameter combined RS was fully developed by combining axillary US findings, clinicopathologic data, and images of breast conventional US. In order to forecast regional LNMs, the Radiomics method was also used in other imaging modalities, such as CT or MRI pictures of several primary cancers, such as bladder or colon cancer, proving that it was a practical approach (40–42). By focusing on the clinical parameter combined RS technique, which can enhance image features with additional information and strengthen the model by restricting the features extracted from pictures, our study produced a superior diagnostic performance than the previous one (24). Furthermore, as compared to other imaging modalities, breast and axillary ultrasounds are routinely used to describe breast lesions and ALN status in patients with suspected breast lesions. They are also less expensive and emit no ionizing radiation (12).

Furthermore, this study demonstrated that the RS was better than radiologists’ assessments in identifying ALNM from both MRI and US scans. There are certain ALNs that are invisible or not picked up by US or breast MRIs. As a remedy for the shortcomings in rural hospitals with limited medical resources, RS of US images is particularly helpful in raising the standard of diagnosis and care.

This study has certain shortcomings that need to be addressed. This is a single-center study, to start. Further multicenter data is required to validate this paradigm prior to future clinical implementation. Second, because it is challenging to identify which lesion will induce ALN metastases and should be entered into the model, individuals with bilateral disease and multifocal breast lesions are not included. Therefore, the level of ALN involvement in patients with solitary breast cancer could only be predicted using the current clinical parameter combination DLR model. Additional research is required to develop alternative models for predicting ALN status in patients with bilateral disease and multifocal breast lesions. Third, individuals are categorized according to their risk of developing breast cancer using gene markers such as BRCA1 and BRCA2 (43). Despite being an intriguing attempt, radiogenomics—which focuses on the connection between genomics and imaging phenotypes—is not yet available. Fourth, the image segmentation technique employed in this study relied on manual delineation; to increase the consistency of our model going forward, automatic delineation of ROIs utilizing DL techniques could take its place. Fifth, we will build a model for SLNM and extra-SLNM prediction, respectively, in the future to address the ambiguity and variability in SLN diagnoses that the current study design is unable to handle.

To sum up, this study demonstrated the clinical use of machine learning methods and introduced the RS that combines important radiomic elements of US images with clinicopathologic traits. In early-stage invasive BC, it can easily and practically identify ALNM patients among several BI-RADS categories and other classifications. It may ultimately lead to a preoperative strategy that directs future clinical therapy.

## Data Availability

The raw data supporting the conclusions of this article will be made available by the authors, without undue reservation.
